# Metabolic Engineering *Escherichia coli* for the Production of Lycopene

**DOI:** 10.3390/molecules25143136

**Published:** 2020-07-09

**Authors:** Zhaobao Wang, JingXin Sun, Qun Yang, Jianming Yang

**Affiliations:** 1 Energy-Rich Compounds Production by Photosynthetic Carbon Fixation Research Center, Shandong Key Lab of Applied Mycology, College of Life Sciences, Qingdao Agricultural University, Qingdao 266109, China; wangzhaobao123@126.com; 2 College of Food Science and Engineering, Qingdao Agricultural University, Qingdao 266109, China; jxsun20000@163.com

**Keywords:** lycopene, the MEP pathway, the MVA pathway, *Escherichia coli*, metabolic engineering

## Abstract

Lycopene, a potent antioxidant, has been widely used in the fields of pharmaceuticals, nutraceuticals, and cosmetics. However, the production of lycopene extracted from natural sources is far from meeting the demand. Consequently, synthetic biology and metabolic engineering have been employed to develop microbial cell factories for lycopene production. Due to the advantages of rapid growth, complete genetic background, and a reliable genetic operation technique, *Escherichia coli* has become the preferred host cell for microbial biochemicals production. In this review, the recent advances in biological lycopene production using engineered *E. coli* strains are summarized: First, modification of the endogenous MEP pathway and introduction of the heterogeneous MVA pathway for lycopene production are outlined. Second, the common challenges and strategies for lycopene biosynthesis are also presented, such as the optimization of other metabolic pathways, modulation of regulatory networks, and optimization of auxiliary carbon sources and the fermentation process. Finally, the future prospects for the improvement of lycopene biosynthesis are also discussed.

## 1. Introduction

Lycopene, a member of the carotenoid family [1], is widely used in food, pharmaceutical, and cosmetic industries because of its potent anti-cancer [2], anti-inflammatory [3], and anti-oxidative activities [4]. Augmentation of lycopene production has become imperative to meet market demand. Currently, lycopene is produced mainly by direct natural extraction, chemical synthesis, and microbial fermentation. Lycopene is also widely found in fruits including tomato, watermelon, guava, and papaya [5,6,7,8], with a concentration of as high as 3–14 mg/100 g in tomatoes [9]. However, the purification process is quite complicated due to numerous carotenoids in the raw materials, and also the extraction method cannot match the large market demand. In addition, lycopene production by chemical synthesis is high-cost, low-yielding, and environmentally unfriendly [10]. Notably, lycopene production by chemical synthesis is banned in the European nations [11]. Therefore, metabolic engineering and synthetic biology for producing lycopene using microorganisms is characterized by high efficiency and environmental friendliness and has been applied as a feasible alternative.

Lycopene, a linear carotenoid with a C40 backbone, is composed of seven isopentenyl diphosphates (IPP) and one dimethylallyl diphosphate (DMAPP), both being its biosynthetic precursors [12,13,14,15]. The production of IPP and its isomer, DMAPP, in vivo via either the 2-*C*-methyl-d-erythritol- 4-phosphate (MEP) pathway [16] or the mevalonate (MVA) pathway is reported [17]. The MEP pathway, present in many bacteria, algae, cyanobacteria, plant chloroplasts, and some eukaryotic parasites [18,19], begins with the condensation of pyruvate and glyceraldehyde 3-phosphate derived from glycolysis [16,20]. In contrast, the MVA pathway, present in most eukaryotes, fungi, plants, archaea, and some bacterial species, can produce IPP and DMAPP using acetyl-CoA as the initial substrate [19,21]. 

Engineered *Escherichia coli* is widely used for the biosynthesis of secondary metabolites and high-value chemicals by metabolic engineering because of its rapid growth and powerful tools to enable genetic manipulation [22]. Moreover, the native MEP pathway present in *E. coli* facilitates the production of terpenoids including lycopene. However, this pathway showed low metabolite flux in metabolic engineering [23], leading to the introduction of the heterogeneous MVA pathway into *E. coli* for lycopene production.

In this review, we summarized the recent advances in lycopene production by the engineered *E. coli* using metabolic engineering strategies. Current investigations on the modification of the two metabolic pathways, MEP and MVA, for lycopene production in *E. coli*, were reviewed. The optimization of other metabolic pathways, modulation of regulatory networks, optimization of auxiliary carbon sources, and the fermentation process were also described. Furthermore, the common challenges, strategies, and prospects for lycopene biosynthesis in metabolically engineered *E. coli* were discussed in this review.

## 2. Metabolic Engineering of Two Major Pathways in *E. coli* for Lycopene Production

### 2.1. The Primary Biosynthetic Pathways for Lycopene Production

**Figure 1:**
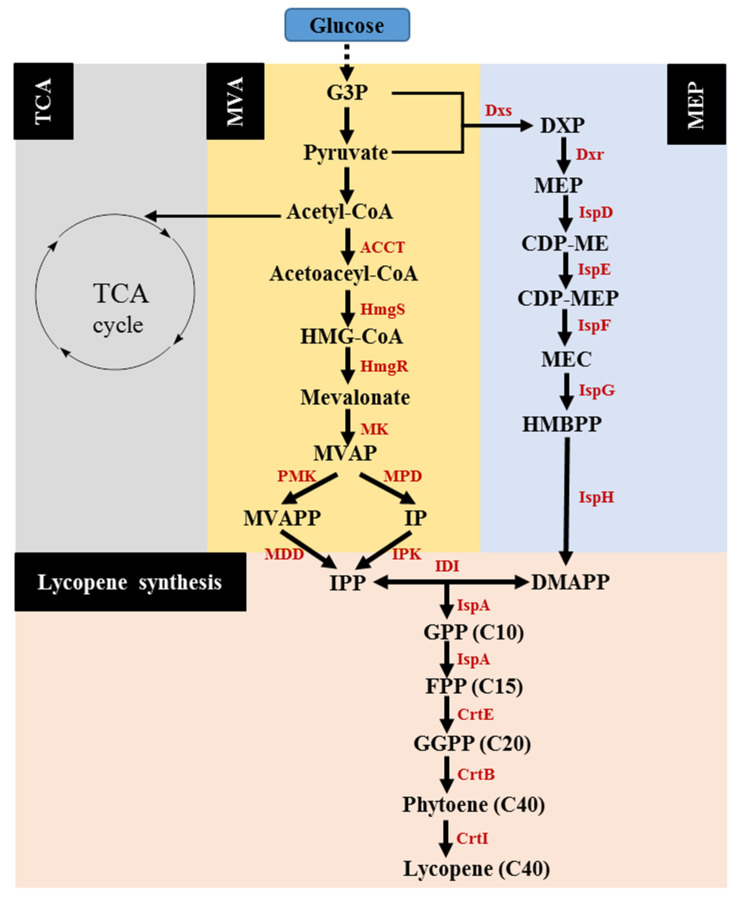
The metabolic pathways for lycopene production. G3P, glyceraldehyde 3-phosphate; DXP, 1-deoxy-d-xylulose-5-phosphate; MEP, methylerythritol phosphate; CDP-ME, 4-diphosphocytidyl-2*C*-methyl-d-erythritol; CDP-MEP, 4-diphosphocytidyl-2*C*-methyl-d- erythritol-2-phosphate; MEC, 2*C*-methyl-d-erythritol-2,4-cyclo-diphosphate; HMBPP, 4-hydroxy-3-methyl-2-(*E*)-butenyl-4-diphosphate; HMG-CoA, 3-hydroxy-3-methylglutaryl-CoA; MVAP, mevalonate-5-phosphate; MVAPP, mevalonate-5-diphosphate; IP, isopentenyl phosphate; IPP, isopentenyl diphosphate; DMAPP, dimethylallyl diphosphate; GPP, geranyl diphosphate; FPP, farnesyl pyrophosphate; GGPP, geranylgeranyl diphosphate; DXS, DXP synthase; DXR, DXP reductoisomerase; IspD, CDP-ME cytidylyltransferase; IspE, CDP-ME kinase; IspF, MEC synthase; IspG, HMBPP synthase; IspH, HMBPP reductase; ACCT, acetoacetyl-CoA thiolase; HmgS, HMG-CoA synthase; HmgR, HMG-CoA reductase; MK, mevalonate kinase; PMK, MVAP kinase; MDD, MVAPP decarboxylase; MPD, MVAP decarboxylase; IPK, IP kinase; IDI, isopentenyldiphosphate isomerase; IspA, FPP synthase; CrtE, GGPP synthase; CrtB, phytoene synthase; CrtI, phytoene desaturase.

The MEP and MVA pathways are the major pathways producing the precursors of lycopene, IPP, and DMAPP. As shown in Figure 1, glyceraldehyde 3-phosphate (G3P) and pyruvate are condensed to form 1-deoxy-d-xylulose-5-phosphate (DXP) by DXP synthase (DXS), followed by the conversion into MEP under the catalyzation of DXP reductoisomerase (DXR or IspC) in the MEP pathway. Further, a series of enzymes, including 4-diphosphocytidyl-2*C*-methyl-d-erythritol (CDP-ME) cytidylyltransferase (IspD), CDP-ME kinase (IspE), 2*C*-methyl-d-erythritol-2,4-cyclo-diphosphate (MEC) synthase (IspF), 4-hydroxy-3-methyl-2-(*E*)-butenyl-4-diphosphate (HMBPP) synthase (IspG), and HMBPP reductase (IspH), successively catalyzes the conversion of MEP into IPP, along with the respective intermediates of CDP-ME, 4-diphosphocytidyl-2*C*-methyl-d-erythritol-2-phosphate (CDP-MEP), MEC, and HMBPP. Subsequently, isopentenyl-diphosphate isomerase (IDI) catalyzes the isomerization of IPP to DMAPP [24,25]. 

The MVA pathway initiates with acetyl-CoA, which is converted into MVA through three reactions, catalyzed by acetoacetyl-CoA thiolase (ACCT), 3-hydroxy-3-methylglutaryl-CoA (HMG-CoA) synthase (HMGS), and HMG-CoA reductase (HMGR), respectively. Subsequently, MVA is converted to mevalonate-5-phosphate (MVAP) catalyzed by mevalonate kinase (MK). The transformation of MVAP into IPP is reported through different pathways. The one in eukaryotes involves two reactions successively catalyzed by MVAP kinase (PMK) and MVAPP decarboxylase (MDD), while the other pathway in archaea harbors two reactions catalyzed by MVAP decarboxylase (MPD) and isopentenyl phosphate kinase (IPK), respectively [26]. After the production of IPP and DMAPP, the condensation reaction between the two intermediates occurs with the formation of geranyl diphosphate (GPP). Further, farnesyl pyrophosphate (FPP) is formed from GPP catalyzed by FPP synthase (IspA). FPP, in turn, is catalyzed by geranylgeranyl diphosphate (GGPP) synthase (CrtE), phytoene synthase (CrtB), and phytoene desaturase (CrtI) to successively form GGPP, phytoene, and finally lycopene [15,27]. The exogenous genes *crtEBI* from various sources can exhibit differential activities when introduced into the host strains. For instance, when different carotenoid genes *crtEBI* were introduced into *E. coli*, higher lycopene yield and cell growth were reached using the genes derived from *Pantoea agglomerans* compared with those of *Pantoea ananatis*. Furthermore, it was identified that *crtE* was responsible for the difference between the engineered *E. coli* strains harboring the *crtEBI* genes of *P. agglomerans* and *P. ananatis*, respectively [15].

To summarize, the sole utilization of the endogenous MEP pathway or co-expression of the MEP and heterogeneous MVA pathways, and the subsequent expression of three key enzymes, CrtE, CrtB, and CrtI [15,27,28,29,30] were the primary biosynthetic route and strategy in the metabolic engineering of *E. coli* for lycopene production. Based on this, further work on the modification of the two major pathways was conducted. Lycopene production via various metabolic engineering optimization strategies in *E. coli* are summarized and listed in Table 1.

### 2.2. Metabolic Engineering of the Endogenous MEP Pathway in E. coli

**Figure 2:**
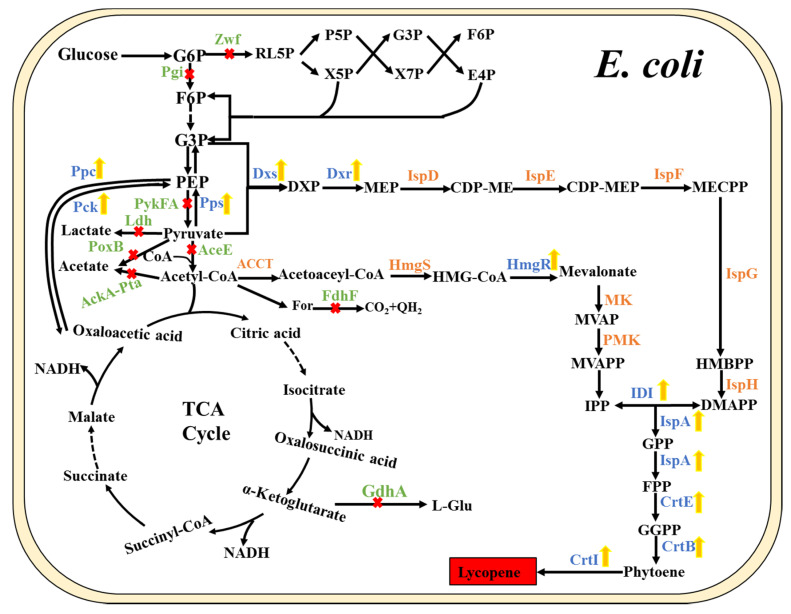
Metabolic engineering strategies of the entire lycopene pathway in *E. coli*. (The yellow arrow represents gene overexpression; the red “×” represents gene knockout or elimination of the pathway). Pps, phosphoenolpyruvate synthase; Pck, PEP carboxykinase; Ppc, PEP carboxylase; Ldh, lactate dehydrogenase; PoxB, pyruvate dehydrogenase; Ack, acetate kinase; Pta, phosphate acetyltransferase; Zwf, glucose-6-phosphate dehydrogenase; Pgi, glucosephosphate isomerase; GdhA, glutamate dehydrogenase; PykFA, pyruvate kinases; AceE, pyruvate dehydrogenase; FdhF, formate dehydrogenase H.

In the early development stages of lycopene production using engineered *E. coli*, the utilization and modification of the endogenous MEP pathway were extensively investigated. Generally, the overexpression of the key enzymes of the MEP pathway is important in lycopene production, especially the major rate-limiting enzymes (DXS, DXR, and IDI) [19]. When the genes *dxs* and *dxr* were overexpressed solely or jointly on different expression vectors using three different promoters and *E. coli* host strains, the highest lycopene yield (22 mg/L) was reached with the arabinose-inducible promoter on a medium-copy plasmid pBAD24 in the *E. coli* XL1-Blue strain [31]. Several key enzymes involved in the biosynthetic pathway were usually co-overexpressed in metabolic engineering. As described, with the co-overexpression of DXS and exogenous carotenoid biosynthetic enzymes, the engineered *E. coli* strain exhibited lycopene production of 1.3 mg/g dry cell weight (DCW) [32]. Similarly, the combinational overexpression of endogenous DXS and IspA and optimal expression of the four exogenous enzymes of IDI, CrtE, CrtB, and CrtI resulted in lycopene production of 5.2 mg/g DCW [13]. Moreover, the co-overexpression of IDI, DXS, IspD, and IspF in the MEP pathway (Figure 2), showed a 6-fold increase in lycopene production (5.39 mg/g DCW) [28]. Besides the overexpression of the rate-limiting enzymes in the pathway, direct evolution and the optimization of ribosome binding sites (RBS) are conducive for the improvement in lycopene production. RBS libraries were utilized to adjust the expression of *dxs*, *idi*, and the *crt* gene operon, leading to an increase in lycopene yield by 32% [27]. Similarly, directed evolution was applied to modulate both the enzymatic expression and specific activity of CrtE. A combination of this and DXS overexpression reached above 45 mg/g DCW of lycopene production [33]. Lv et al. constructed a lycopene-indicated high-throughput screening method for isoprene production by performing directed co-evolution of the key enzymes (DXS, DXR, and IDI) of the MEP pathway. The result indicated a potential for pathway optimization in lycopene production [34]. In addition, when the expression of *dxs*, *idi*, and *crt* operon genes was modulated using the RBS library, a significant improvement in lycopene yield was observed [27].

Most research has been focused on improving activities of the desired enzymes via conventional strategies, such as overexpression, direct evolution, and RBS optimization for lycopene production using the MEP pathway. However, fewer studies have been performed to investigate the regulatory mechanisms of the metabolic pathway. Therefore, further understanding of the regulatory mechanism of the MEP pathway and the combination of different strategies for lycopene production in *E. coli* need to be explored in the future.

### 2.3. Metabolic Engineering of the Heterogeneous MVA Pathway

Although the MEP pathway is natively present in *E. coli* [16], the utilization of this pathway has not shown high efficiency and exhibits low metabolite flux [23]. In contrast, the MVA pathway, an alternative pathway for lycopene production, is more energy-saving than the MEP pathway [30], and its introduction paved a new way for the formation of IPP from acetyl-CoA, resulting in a high-efficient lycopene production in *E. coli*. The MVA pathway is composed of the upper and lower pathway, separate from MVA [53]. When the whole MVA pathway from acetyl-CoA to IPP was engineered into *E. coli*, lycopene production increased by over 2-fold compared with the control strain [35]. Similarly, when only the lower pathway was employed, and mevalonate was supplied as a substrate, a significant increase in lycopene production was also observed [54]. These results indicated that the introduction of either the complete or the partial MVA pathway could reach an effective lycopene yield when a carbon source or carbon source with mevalonate were supplied, respectively, both through increasing lycopene precursors IPP and DMAPP. The heterologous MVA pathway was transferred into *E. coli* DH5α, combined with the overexpression of a type 2 IDI from *Bacillus licheniformis* significantly elevating lycopene production [36]. However, these studies were all implemented at the shake-flask fermentation level, not yet scalable for industrial production. Therefore, Zhu et al. adopted a new, targeted engineering strategy to reconstitute the MVA pathway in *E. coli* and establish a highly efficient platform which was employed for lycopene production. The fed-batch fermentation process was scaled up to 100 L, reaching 1.23 g/L of lycopene concentration with a maximum productivity of 74.5 mg/L/h [47]. In addition, Miguez et al. had conducted metabolomics analysis to reveal the toxic effects of lycopene production and the metabolic differences caused by induction time variation of the MVA pathway in the engineered *E. coli* strain. They reported that overnight induction of the MVA pathway was toxic to cells, which could recover if the lycopene pathway was not heterologously expressed simultaneously. Further, they validated that the intermediate homocysteine could contribute to the growth inhibition and the antagonistic effect between the mevalonate and lycopene pathways, resulting in the homocysteine-induced toxicity in lycopene production. This work indicated that metabolomics would be beneficial to reveal the mechanisms of the metabolite toxicity, and subsequently help to improve the metabolic engineering for the biosynthesis of carotenoid [37].

The introduction of the MVA pathway into *E. coli* caused dramatic improvement in lycopene production and could serve as a platform for the production of carotenoid compounds. This was attributed to the better elucidation of the MVA pathway than the MEP pathway. Moreover, the endogenous MEP pathway could be influenced by native regulation. In contrast, the introduction of the exogenous MVA pathway could play its role without regulation. However, because of the common intermediates (IPP and DMAPP), the two pathways are not totally independent, which provides potential optimization strategies for balancing two different pathways for carotenoid compounds production, including lycopene.

## 3. Optimization of Other Metabolic Pathways to Enhance Lycopene Production

In metabolic engineering, inhibition or even knockout of the competitive pathway of intermediate products, and elimination of potential bottlenecks in the upstream pathways not only reduces the generation of by-products but also increases the yield of the target products. Meanwhile, the enhancement of some metabolic pathways is also adopted to modulate the metabolite flux in the synthesis of the target products by supplying more precursors or intermediates. Thus, a rational design strategy of metabolic pathways is of great potential to increase the yield of the target products.

As per the description of the MEP pathway, the initial precursors, G3P and pyruvate, are condensed into DXP in equal amounts. The unbalance between the two precursors can reduce the synthetic efficiency, making it necessary to maintain the balance by modulating the metabolic pathways based on a rational design strategy. As is known, the conversion from phosphoenolpyruvate to pyruvate [55], an essential step in the transformation of G3P to pyruvate, is an irreversible reaction [20] inhibiting the inter-conversion between G3P and pyruvate. Consequently, a new circuit bypassing this irreversible step was reconstructed. This was achieved by deleting pyruvate kinases-I and -II (Pyk-I and-II) to cut off the direct conversion of PEP to pyruvate, and overexpressing Ppc and Pck to introduce the bypass pathway between PEP and pyruvate via the oxaloacetate and TCA cycle (Figure 2). Meanwhile, the PEP synthase (Pps) converting pyruvate to PEP was also overexpressed. Thus, lycopene production was significantly increased with the rational reconstruction of the metabolic pathways from G3P to pyruvate [20]. 

Similarly, the inactivation of the competing pathways at acetyl-CoA and pyruvate nodes was applied to divert more carbon flux to the precursor IPP, including the deletion of the acetate and lactate production pathways. As a result, the engineered strain with the elimination of the acetate pathway accumulated more lycopene than the control strain [35]. Generally, metabolic engineering of pathways was mainly referred to the manipulation of genes directly connected with the product-synthesizing pathway. At the same time, indirectly related genes could also influence the synthesis of target products. For example, the deletion of the *zwf* gene encoding glucose-6-phosphate dehydrogenase (G6PD) (Figure 2), which controls the entry of carbon into the pentose phosphate pathway, resulted in the increased carbon fluxes in the Embden–Meyerhof–Parnas (EMP) pathway involving G3P and pyruvate, and indirectly led to an increase in lycopene production [28].

Identification of the genes directly or indirectly related to the production of the target products in metabolic engineering is of utmost importance. The elimination or overexpression of these genes can affect metabolic progress, either by redistributing the metabolic precursors or rewiring regulatory networks. Previous research on metabolically engineering *E. coli* for lycopene production employed an artificial phenotypic screening system to identify the genes affecting lycopene formation. As a result, multiple genes, including two unknown genes *elb1* and *elb2*, were identified that might be involved in the early reactions in lycopene synthesis. In more detail, the regulator encoded by *elb2* could regulate the biosynthesis of ubiquinone, including early steps of isoprenoid biosynthesis [56]. Alper et al. performed a genome-wide stoichiometric flux balance analysis to explore potential genes impacting the whole network properties and cellular phenotype. Consequently, seven single and multiple stoichiometric gene deletion mutants were obtained with increased lycopene production compared with the parental strain. Mainly, the triple knockout mutant of *gdhA/aceE/fdhF* (encoding NADP-specific glutamate dehydrogenase, pyruvate dehydrogenase, and formate dehydrogenase, respectively) exhibited a nearly 40% increase in lycopene yield (Figure 2). By exploring the potential reasons, it was found that *gdhA* deletion could increase the availability of NADPH, the knockout of *aceE* would presumably improve carbon flux to pyruvate and formate, and further deletion of *fdhF* might redirect the formate flux back to pyruvate, resulting in the increase of lycopene production [39]. Furthermore, transposon mutagenesis was utilized to identify combinatorial genetic targets for deletion to increase either the cofactor or precursor supply, resulting in the enhancement of lycopene yield. All the validated deletions were directly or indirectly related to cofactor production or metabolic flux [38]. Based on transposon mutagenesis and screening, the Δ*hnr* (aspartokinase/homoserine dehydrogenase) Δ*yliE* (di-GMP phosphodiesterase) mutant was obtained that significantly improved lycopene production. The Hnr protein could function as RpoS degradation, which is important for carotenoid production in *E. coli*. Moreover, the absence of the hypothetical protein YliE could positively influence lycopene production only in the Δ*hnr* background [40]. Subsequently, a two-dimensional gene target search of systematic and combinatorial approaches was developed to identify the overexpression targets and the knockout targets, respectively. More than 40 engineered strains were constructed, and the corresponding lycopene production was detected, combining overexpression with the knockout of the target genes with the highest lycopene yield being 16 mg/g DCW. These mutants involved kinds of genes referring to the synthetic pathway and regulation, and the lycopene of the most mutant strains has been limited by regulatory or metabolic barriers [48]. The adoption of a multi-dimensional search could thus help explore extensive mutant phenotypes. As a result, effective tools and strategies are necessary for identifying potential genes related to product yield in metabolic engineering at a global level. Moreover, the approach used to tune the genetic control of a single gene and modify multiple genes simultaneously also has been developed, including a functional promoter library [57], global transcription machinery engineering [58], engineered global regulators [42], and a functional RBS library [27]. The improvement of these tools and strategies would be beneficial for the optimization of the metabolic landscapes and the construction of effectively engineered strains.

**Figure 3:**
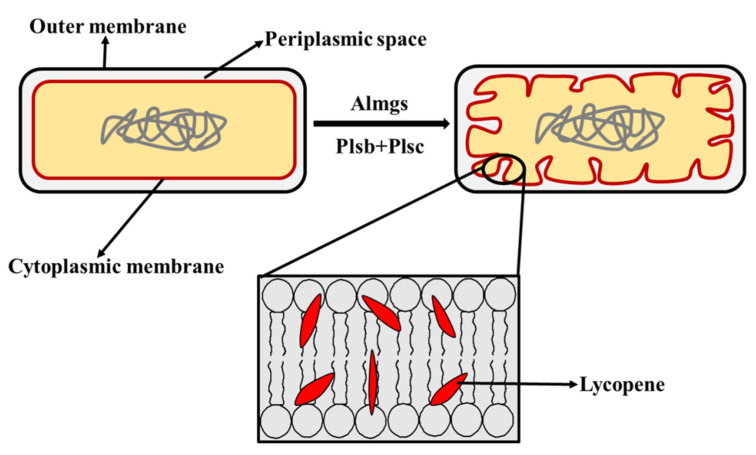
Diagram of membrane engineering strategy to increase the lycopene production in *E. coli*. Almgs, membrane-bending protein; Plsb, glycerol-3-phosphateacyltransferase; Plsc, 1-acylglycerol-3-phosphate-acyltransferase.

Besides the modulation of the metabolite flux, a specific strategy such as membrane engineering plays a role in improving the lycopene yield. As shown in Figure 3, when Almgs (a membrane-bending protein) and two proteins related to the membrane-synthesis pathway, Plsb (glycerol-3-phosphate acyltransferase) and Plsc (1-acylglycerol-3-phosphate-acyltransferase), were overexpressed, the cells produced sufficient intracellular membrane vesicles and thus provided more space, and the amount of the membrane component, glycerophospholipids, was increased. Finally, 36.4 mg/g DCW of lycopene was accumulated in the cell membranes [49]. This novel membrane engineering strategy could be further explored for the synthesis of a wide range of hydrophobic products.

In addition, the precursors of lycopene, IPP and DMAPP, were biosynthesized via the MEP or MVA pathway, during which they need NADPH and ATP [19]. Consequently, another critical engineering strategy for enhancing lycopene production is to provide enough NADPH and ATP to the entire metabolic pathway. In another study, the expression of genes encoding a-ketoglutarate dehydrogenase, succinate dehydrogenase, and transaldolase B was modulated to increase NADPH and ATP, resulting in a significant increase in the lycopene yield [27]. 

## 4. Engineering Regulatory Networks to Enhance Lycopene Production

As mentioned above, most studies on lycopene production were focused on the overexpression of the key rate-limiting enzymes, and the elimination or inactivation of the competitive branch pathway. The regulation of metabolic pathways could be used to reprogram the metabolic genes to improve the yield of the target products by eliminating metabolic imbalance. 

**Figure 4:**
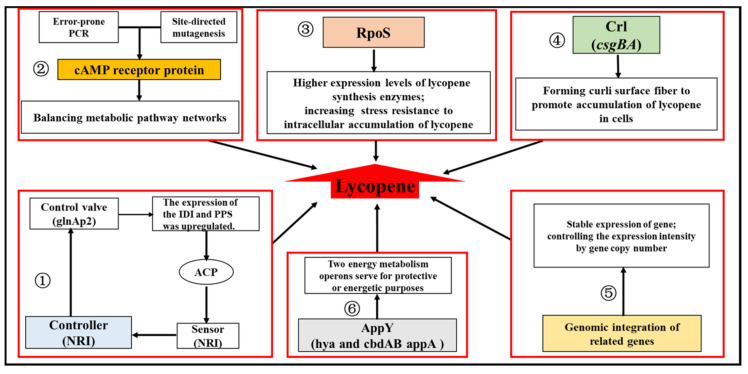
Summary of the strategies for engineering the regulatory networks to enhance lycopene production in *E. coli*. (1) Acetyl phosphate (ACP), as an indicator of glucose flux, was set as a signal of the two-component regulon Ntr, to regulate the expression of *idi* and *pps* for lycopene biosynthesis. (2) Transcriptional engineering on the global regulator cAMP receptor protein (CRP) was conducted by using error-prone PCR and site-directed mutagenesis, to subtly balancing the whole metabolic pathway networks for improving the lycopene yield. (3) RpoS regulates the transcription of genes induced at the stationary phase and energy metabolism. (4) Crl regulated the expression of *csgBA* for curli surface fiber formation to promote the accumulation of lycopene in cells. (5) Genomic integration of related genes made the expression stable and controlled the expression intensity by gene copy number. (6) The AppY transcriptional regulator was relative to anaerobic energy metabolism.

The phosphorylated response regulator NRI (*glnG* product) included in the two-component system Ntr could activate transcription from the *glnAp2* promoter by binding to its cognate binding sites on the DNA. Further, NRI itself is capable of sensing the level of acetyl phosphate (ACP), an indicator of glucose flux. Therefore, an artificially engineered *glnAp2* promoter containing NRI binding sites and the core *glnAp2* promoter was constructed to serve as a control valve that controls gene expression according to the ACP level. Then, this control valve was adopted to regulate the expression of IDI and Pps, which have been identified to control the flux to the final product, and the balance between pyruvate and G3P. The introduction of this regulatory circuit resulted in the full utilization of the excess carbon flux for the enhancement of lycopene production and bypassing the toxic product, acetate [41]. Moreover, transcriptional engineering of the global regulator cAMP receptor protein (CRP) was conducted using an error-prone PCR and site-directed mutagenesis to subtly balance the whole metabolic pathway networks to improve lycopene yield. The mutant strain, MT-1, with the engineered CRP encoded by a mutant gene (mcrp26) showed a higher lycopene production (18.49 mg/g DCW) compared with the original strain. Besides, the differential expression of the global genes between the MT-1 mutant and wild type was also explored, in which the genes of *pfkA*, *fbaA*, and *ispG* involved in the lycopene biosynthetic pathway were up-regulated. Thus, it helped reveal the possible mechanism for the improvement in lycopene production caused by the engineered CRP. As explored, in mcrp26, residue D8 (Asp) had been mutated into V (Val), which belongs to the N-proximal cAMP-binding domain, altering the cAMP-binding capacity. This influenced the CAP-dependent promoters, which raised the differential expression of the above genes related to lycopene production [42]. In addition, the modulation of global regulatory proteins RpoS (Sigma S factor), AppY (transcription activator for genes related to anaerobic energy metabolism), and Crl (transcriptional regulator of *csgBA* for curli surface fiber formation) also enhanced lycopene production by regulating the expression of lycopene synthesis enzymes and energy metabolism operons [59], or the hydrophobic interaction between curli fiber molecules and lycopene [29,48,60]. Another study was performed to disclose the mechanisms involved in the differences in the lycopene production and MEP pathway flux of six *E. coli* host strains through systems analysis including genetic complementation, quantitative sequential windowed acquisition of all theoretical fragment ions (SWATH) proteomics, and biochemical analysis. It revealed that RpoS could help accumulate lycopene by decreasing oxidative stress in the growth stationary phase, which reduced the degradation of lycopene to its colorless oxidation—and cleavage products [61]. These strategies for engineering the regulatory networks to enhance lycopene production in *E. coli* have been summarized in Figure 4. Thus, the engineered regulators controlling the gene expression gave rise to a significant potential for the regulatory design of metabolic pathways in *E. coli* for the production of lycopene and other biochemicals.

Although the biochemistry properties of the MVA pathway have been comprehensively revealed and extensively utilized for the industrial production of isoprenoids, few applications of the regulation of the MVA pathway have been explored in lycopene biosynthesis. Moreover, the regulatory mechanisms of the MEP pathway are also less studied. Thus, the adoption of the regulation of the MVA pathway and the in-depth understanding of the MEP pathway are necessary for further optimization on improving lycopene production in *E. coli*.

## 5. Optimization of Auxiliary Carbon Source and Fermentation Modes

Besides the optimization of regulatory networks in a metabolic pathway, the supply of an appropriate carbon source is also an essential factor for improving synthetic efficiency, thereby reducing the production costs at the industrial level. In the biosynthesis of lycopene, glycerol or glucose are usually utilized as the primary carbon source [36,43,45,47,54,62]. Some researchers explored the effect of an auxiliary carbon source on lycopene production in engineered *E. coli*. When glycerol was used as the primary carbon source, the synergistic effect of the auxiliary carbon sources, glucose, and l-arabinose was related to the endogenous metabolism in *E. coli* and the stimulation on the exogenous MVA pathway [43]. When glucose, fructose, glycerol, or arabinose were supplied as an auxiliary carbon source, respectively, to the LB medium for lycopene production, 6 g/L fructose exhibited the highest lycopene yield [12]. Citrate was verified to be a positive auxiliary carbon source for enhancing lycopene production, indicating that the citrate pathway might be responsible for accumulating more isoprenoid in engineered *E. coli* [36]. Similarly, in another study, the MVA lower pathway was introduced into *E. coli*, in which glycerol was supplied as the carbon source with addition of mevalonate, and Tween 80 was added to prevent clump formation, resulting in a significant increase in lycopene production [54]. Moreover, an engineered *E. coli* strain introduced with the fatty acid transport system was capable of utilizing free fatty acids or waste cooking oil to produce lycopene, with the highest yield of 94 mg/g [44].

Optimization of the fermentation process is a traditional and direct strategy for improving lycopene production. Traditional fermentation that includes shake-flask and fed-batch fermentations is usually applied (Table 1). Besides this, various fermentation optimization strategies have been conducted. For instance, high cell density fermentation was performed in two lycopene-producing *E. coli* strains to explore the effect of fermentation parameters on lycopene production. Results demonstrated that high oxygen levels and pH values were critical for increasing the lycopene yield. The importance of oxygen, growth rate, and glutamate flux on lycopene production thus indicated the potential of stoichiometric analysis in optimizing the fermentation strategy [45]. In another study, considering the increased carotenoid yield and productivity at 25 °C in contrast to those at 37 °C [35,63], a temperature-shift culture method (37→25 °C) was adopted to further augment lycopene production [62]. Optimizing the culture conditions of the recombinant *E. coli* 99DH cultivated under exposure to light in 2YT medium (1.6% tryptone, 1% yeast extract, 0.5% NaCl), supplemented with glycine addition, exhibited an increased lycopene production rate by 76% [46].

## 6. Other Engineering Strategies for Lycopene Production

Besides the conventional engineering approaches adopted to enhance lycopene production in *E. coil*, some novel engineering strategies have been explored. For instance, the enhancement of mRNA stability by varying the mRNA secondary structures was adopted to modulate the metabolic flux to improve lycopene production [64]. Moreover, during the amplification of the fermentation process, it is essential to decrease the requirement of complex media and antibiotics, and the burden caused by exogenous plasmids, as well as to maintain the stability of the engineered strain, based on which chromosomal integration is applied to introduce lycopene synthesis genes into the *E. coli* chromosome [50]. A multiplex automated genome engineering for large-scale programming had been used to optimize the MEP pathway in *E. coli* for lycopene production [51]. Coussement et al. developed a new combinatorial multi-gene pathway assembly scheme based on single-strand assembly (SSA) methods and Golden Gate assembly, and it was adopted to optimize the lycopene biosynthetic pathway, resulting in lycopene production of 448 mg/g DCW [52].

## 7. Discussion and Future Perspectives

In summary, all these efforts on exploring various engineering strategies have facilitated an increase in lycopene production by metabolically engineering *E. coli*. However, there is still much room for improvement for lycopene yield in engineered *E. coli*. The following perspectives could be focused on in future research. First, the combination of the endogenous MEP pathway and the heterogeneous MVA pathway should be further investigated. The mechanisms of the cross-talk between the two pathways should also be uncovered. Second, the regulatory mechanism of the MEP pathway has not been well understood, giving rise to the potential of exploring regulatory engineering to enhance the biological production of lycopene. Third, the compartmentalization of the lycopene biosynthetic pathway should be paid more attention to, which is beneficial to eliminate the cytotoxicity of IPP and DMAPP as well as enhance the compartmental synthesis efficiency. Accumulation of the target products results in gradual cellular pressure of tolerating the target products, thus requiring the exploration of new strategies to resolve this issue, such as adaptive evolution, membrane engineering and efficient extraction methods. Finally, more concerns are given to the modification of upstream metabolic pathways to increase lycopene production, while the optimization of the downstream fermentation process is ignored. Therefore, more attempts should be made to the process engineering of the fermentation, including optimization of the fermentation mode and process parameters, in situ product recovery (ISPR) processes, and so on. Actually, there are numerous research directions in engineered *E. coli*, showing a promising prospect for lycopene production and other biochemicals.

## Figures and Tables

**Table 1:** Summary of the metabolic engineering optimization strategies used for the production of lycopene in *E. coli*.

Major Methods	Optimization Strategies	Yield/Titer	Culture Conditions	References
Overexpression of rate-limiting enzymes	Comparison of *crtEBI* genes from different strains	59 mg/L	-	[15]
	Knockout of *zwf*; overexpression of *idi*, *dxs* and *ispD*, *ispF*	7.55 mg/g DCW	Shake-flask fermentation	[28]
	Overexpression of *crtE*, *crtB*, *crtI*, *ipi*, *dxs*	5.2 mg/g DCW	Shake-flask fermentation	[29]
	Overexpression of *dxs*, *dxr*	22 mg/L	Shake-flask fermentation	[31]
	Overexpression of *dxs*	1.33 mg/g DCW	Shake-flask fermentation	[32]
	The co-expression of *appY*, *crl*, and *rpoS* with *dxs*	4.7 mg/g DCW	-	[29]
Directed evolution	Directed evolution of GGPP synthase	45 mg/g DCW	Shake-flask fermentation	[33]
	Directed co-evolution of *dxs*, *dxr* and *idi*	0.65 mg/L	-	[34]
Whole pathway engineering	Expression of the MVA pathway	4.28 mg/L	Shake-flask fermentation	[35]
	Type 2 IDI; heterologous MVA pathway	198 mg/g DCW	Shake-flask fermentation	[36]
	Heterologous expression of the MVA pathway	-	Shake-flask fermentation	[37]
Removal of competing pathways	Δ*gdhA*, Δ*aceE*, Δ*ytjC* (*gpmB*), Δ*fdhF*	18 mg/g DCW	Batch shake-flask cultivations	[38]
Pathway balancing	Combination of gene knockout and overexpression	2.5 mg/g DCW	-	[20]
	Genome-wide stoichiometric flux balance analysis; genes knockouts	6.6 mg/g DCW	Shake-flask fermentation	[39]
	Gene knockout (Δ*hnr*, Δ*yliE*)	-	Shake-flask fermentation	[40]
Regulatory engineering	Ntr regulon, stimulated by excess glycolytic flux through sensing of ACP	0.16 mg/L/h	Shake-flask fermentation	[41]
	Engineering of the cAMP receptor protein (CRP)	18.49 mg/g DCW	Batch fermentation	[42]
Optimization of carbon sources	Auxiliary carbon source optimization	1050 mg/L	Baffled flask fermentation	[12]
	Supplementing auxiliary carbon sources	40 mg/L/h	Fed-batch culture	[43]
	Fermentation with fatty acids or waste cooking oils	94 mg/g DCW	Fed-batch fermentation	[44]
Optimization of fermentation	High cell density fermentation	220 mg/L	Batch fermentation	[45]
	Different types of plasmid expression; optimization of fermentation conditions	67 mg/g DCW	Shake-flask fermentation	[46]
Targeted engineering	Targeted engineering; targeted proteomic and intermediate analysis	1.23 g/L	Fed-batch fermentation	[47]
	Two-dimensional search for gene targets	16 mg/g DCW	Shake-flask fermentation	[48]
Cofactor engineering	Modulating supply of NADPH and ATP; overexpression of *dxs*, *idi* and the *crt* gene operon	50.6 mg/g DCW	Fed-batch fermentation	[27]
Membrane engineering	Membrane engineering; overexpression of *plsb*, *plsc* and *dgka*	36.4 mg/g DCW	Shake-flask fermentation	[49]
Genome engineering	Synthesis genes were integrated into chromosome	33.43 mg/g DCW	Shake-flask fermentation	[50]
	Large-scale programming used to optimize the MEP pathway	9 mg/g DCW	-	[51]
	A new combinatorial multi-gene pathway assembly scheme	448 mg/g DCW	-	[52]
